# Methylprednisolone blocks interleukin 1 beta induced calcitonin gene related peptide release in trigeminal ganglia cells

**DOI:** 10.1186/s10194-016-0609-x

**Published:** 2016-03-01

**Authors:** Lars Neeb, Peter Hellen, Jan Hoffmann, Ulrich Dirnagl, Uwe Reuter

**Affiliations:** Department of Neurology and Experimental Neurology, Charité Universitätsmedizin Berlin, Charitéplatz 1, 10117 Berlin, Germany; Department of Neuroradiology, Universitätsmedizin Göttingen, Robert-Koch-Straße 40, 37075 Göttingen, Germany; Department of Systems Neuroscience, University Medical Center Hamburg-Eppendorf, Martinistrasse 52, D-20246 Hamburg, Germany

**Keywords:** Cluster headache, Migraine, Trigeminal ganglia cells, Calcitonin gene related peptide, Methylprednisolone, Metoprolol, Interleukin-1β, Prostaglandin E_2_

## Abstract

**Background:**

Methylprednisolone (MPD) is a rapid acting highly effective cluster headache preventive and also suppresses the recurrence of migraine attacks. Previously, we could demonstrate that elevated CGRP plasma levels in a cluster headache bout are normalized after a course of high dose corticosteroids. Here we assess whether MPD suppresses interleukin-1β (IL-1β)- and prostaglandin E_2_ (PGE_2_)-induced CGRP release in a cell culture model of trigeminal ganglia cells, which could account for the preventive effect in migraine and cluster headache. Metoprolol﻿(MTP), a migraine preventive with a slow onset of action, was used for comparison.

**Methods:**

Primary cultures of rat trigeminal ganglia were stimulated for 24 h with 10 ng/ml IL-1β or for 4 h with 10 μM PGE_2_ following the exposure to 10 or 100 μM MPD or 100 nM or 10 µM MTP for 45 min or 24 h. CGRP was determined by using a commercial enzyme immunoassay.

**Results:**

MPD but not MTP blocked IL-1β-induced CGRP release from cultured trigeminal cells. PGE_2_-stimulated CGRP release from trigeminal ganglia cell culture was not affected by pre-stimulation whether with MPD or MTP.

**Conclusion:**

MPD but not MTP suppresses cytokine (IL-1β)-induced CGRP release from trigeminal ganglia cells. We propose that blockade of cytokine mediated trigeminal activation may represent a potential mechanism of action that mediates the preventive effect of MTP on cluster headache and recurrent migraine attacks.

## Background

The trigeminal system and the neuropeptide calcitonin gene-related peptide (CGRP) are key players in migraine and cluster headache pathophysiology. Activation of perivascular trigeminal nerves within the meninges causes the release of CGRP [1, 2]. CGRP plasma levels were elevated during migraine and cluster headache attacks and effective attack treatment led to the normalization of CGRP levels [3, 4]. The release of CGRP contributes to vasodilatation, neurogenic inflammation, transmission of pain signals and central sensitization [5]. These mechanisms seem to be of significance in migraine pathophysiology and might also be involved in cluster headache pathophysiology.

CGRP plasma levels may also serve as biomarkers for primary headaches. Patients with episodic and chronic migraine demonstrate elevated CGRP plasma levels between attacks [6]. Recently, we could demonstrate that CGRP plasma levels are elevated interictally in episodic cluster headache patients in the bout and that these levels are reduced after short term prophylaxis with corticosteroids [7]. We hypothesized that elevated CGRP plasma levels in a cluster bout might represent a hyperactive state of the trigeminal nervous system. Suppression of trigeminal hyperactivity could be a consequence of corticosteroid therapy, which in turn leads to the suppression of cluster headache attacks. However, our study could not exclude that altered CGRP levels were rather a consequence than the cause of the reduced attack frequency.

Previously, we observed in cultured trigeminal ganglia cells that interleukin 1β (IL-1β) and prostaglandin E_2_ (PGE_2_) induce CGRP release in a cyclooxygenase-2 dependent pathway [8]. Cytokines and especially IL-1β have been linked to migraine [9, 10] and cluster headache [11] and an involvement of pro-inflammatory cytokines in the pathophysiology of primary headaches is probable.

To determine whether corticosteroids may influence trigeminal activation directly, we studied the effects of corticosteroids on CGRP release in this trigeminal ganglia cell model using IL-1β and PGE_2_ for stimulation. In addition to short-term cluster headache prophylaxis methylprednisolone (MPD) is also used to abort a migrainous state or to prevent the recurrence of migraine attacks [12]. Therefore, we compared the effects of MPD, a drug with rapid onset of action, with the slowly acting migraine preventive metoprolol (MTP) on CGRP release in this model.

## Methods

### Animals

We used 3-day-old male and female Sprague Dawley rats (Charles River, Sulzheim, Germany). All animals were kept under standard laboratory housing conditions with a 12 h light–dark cycle and with an adult female Sprague Dawley rat (Charles River, Sulzheim, Germany) with free access to food and water. For cell culture procedures newborn animals were anaesthetized with an isoflurane vaporizer (4 %) and decapitated. All animal work was carried out in accordance with the European Communities Council Directive of 24 November 1986 (86/609/EEC) regarding the care and use of animals for experimental procedures. The sacrifice of the rats and extraction of their brains was approved by and reported to the Landesamt für Gesundheit und Soziales Berlin (LaGeSo; T0322/96).

### Cell culture

Trigeminal ganglia cell culture was established as previously described by our group [8]. In brief, trigeminal ganglia were dissected from 3 day old male and female Sprague Dawley rats (Charles River, Sulzheim, Germany). The cells were incubated for 90 min at 37 °C in 10 ml dissociation medium (modified eagles medium; Biochrom, Berlin, Germany; with 10 % bovine serum, 10 mM HEPES, 44 mM glucose, 100 U penicillin + streptomycin, 2 mM glutamine, 100 IE insulin/l) containing collagenase/dispase (final concentration 100 μg/ml) (Boehringer Mannheim, Germany), rinsed twice with phosphate buffered saline (PBS) 0.1 M and again incubated with trypsin/EDTA (0.05 %/0.02 % w/v in PBS) for 30 min for dissociation. Subsequently, cells were rinsed twice with PBS and once with dissociation medium, dissociated by Pasteur pipette and pelleted by centrifugation at 2100 x g for 2 min at 21 °C. After suspension in starter medium (Invitrogen, Karlsruhe, Germany) plus 1 % penicillin/streptomycin, 0,25 % L-glutamine, 2 % B27-supplement, 0,1 % 25 mM glutamate, 2.5 mM calcium chloride and 100 ng/ml nerve growth factor-β, cells were plated in 24 well plates and filled to 500 μl with starter medium at a density of 0.5 x 10^−6^ cells/cm^2^ (equates approximately 2 ganglia/well). Wells were pretreated by incubation with poly-l-lysin (5 % w/v in PBS) for 90 min at 4 °C, then rinsed with PBS, followed by incubation with coating medium (dissociation medium with 1 % w/v collagen G) for 90 min at 37 °C in the incubator. After that, the wells were rinsed twice with PBS and filled with starter medium in which cells were seeded. Cytosine arabinoside (final concentration 10 μM; Sigma Aldrich, Munich, Germany) was added at day 1 and day 3 to minimize growth of non-neuronal cells. Cultures were kept at 37 °C and 5 % CO_2_ and fed with neurobasal medium + B27 medium every second day by replacing 50 % of the medium. Condition of cultures was assessed by light microscopy. Stimulation experiments were performed on day 6.

### CGRP determination by enzyme immunoassay

After 6 days in culture the medium was gently removed and replaced with fresh medium without nerve growth factor to exclude effects of nerve growth factor on protein release. 1 h later cells were stimulated for 24 h with IL-1β (10 ng/ml), 4 h with PGE_2_ (10 μM) or equal volume of vehicle (PBS 0.1 M). For inhibition studies cells were pre-incubated with MPD (10 μM or 100 μM), MTP (100 nM or 10 μM) or PBS 45 min or 24 h prior to simulation with vehicle (PBS), IL-1β or PGE_2_. Immediately before the stimuli 50 μl supernatant of each well were removed to assess baseline CGRP levels. At the end of the stimulation supernatants of two dishes were pooled and used for CGRP determination with a specific CGRP enzyme immunoassay (SPIbio, Montigny le Bretonneux, France) as recommended by the manufacturer. For each experiment, one set of wells was treated with 60 mM KCl to determine the responsiveness of the cultures to an established depolarizing stimulus [13]. Cultures that exhibited a response less than 2-fold on CGRP release after the depolarizing stimulus were not analyzed. CGRP release was determined in pg/ml as absolute increase over baseline values in the corresponding two wells (CGRP levels after stimulation – baseline CGRP levels before stimulation). All samples were measured in duplicates. Each experimental condition was repeated in at least seven independent experiments.

### Statistical analysis

Due to small sample size nonparametric statistics were used. Differences of CGRP values between groups were analysed with the Kruskal-Wallis H test. If this test showed statistical significance pairwise comparison was performed using the Mann–Whitney U test. Resulting *p*-values were adjusted for multiple comparisons using the Bonferroni-Holm method. Corrected *p* < 0.05 was considered statistically significant. All statistical tests were performed with the SPSS 20 statistical software (SPSS, Chicago, IL, USA). Data are shown as mean ± standard error of the mean (SEM).

## Results

In a first step we investigated the effect of MPD on basal and stimulated CGRP release in cultures of rat trigeminal ganglia cells. Cultures were pretreated with vehicle (PBS) or MPD (10 or 100 μM) for 45 min followed by stimulation with PBS or IL-1β (10 ng/ml) for 24 h.

**Fig. 1:**
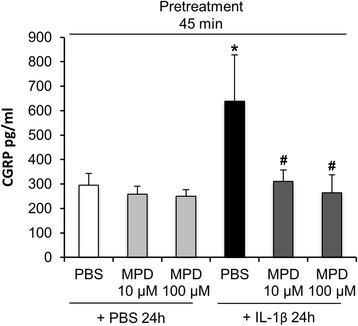
Pretreatment with methylprednisolone (MPD) suppressed IL-1β-stimulated CGRP release in trigeminal ganglia cell culture. Kruskal-Wallis test followed by Mann–Whitney U test with *p*-values adjusted for multiple comparisons using the Bonferroni-Holm method was used to determine significant differences. IL-1β (10 ng/ml) but not vehicle stimulation (PBS) resulted in significantly enhanced CGRP levels in the supernatant of cultured trigeminal ganglia cells after 24 h (*p = 0.031 vs. vehicle). Exposure to MPD 10 μM or 100 μM 45 min prior to stimulation with IL-1β blocked CGRP release significantly compared to pre-treatment with PBS (# *p* = 0.022 (10 μM) and *p* = 0.012 (100 μM). CGRP levels are shown in mean pg/ml ± SEM, *n* = 9

A Kruskal-Wallis H test showed that there was a statistically significant difference in CGRP levels between the different stimulations (χ^2^ (3) = 10.270, *p* = 0.016). Pairwise comparison using the Mann–Whitney U test with correction for multiple comparison (Bonferroni-Holm method) revealed that stimulation of trigeminal ganglia cells with IL-1β led to a significantly increased CGRP release compared to control (PBS) (IL-1β: 638 ± 189 SEM pg/ml vs. PBS: 295 ± 48 SEM pg/ml; *n* = 9; *p* = 0.031). Administration of 10 μM or 100 μM MPD into the culture (45 min before stimulation with IL-1β) led to a statistical significant suppression of IL-1β-stimulated CGRP release (310 ± 47 SEM pg/ml; *p* = 0.022 (10 μM) and 264 ± 74 SEM pg/ml; *p* = 0.012 (100 μM); *n* = 9). In contrast, pretreatment of cultures with MPD for 45 min itself without adding IL-1β did not significantly change the amount of CGRP release in controls (PBS exposure solely) (Fig. 1).

**Fig. 2:**
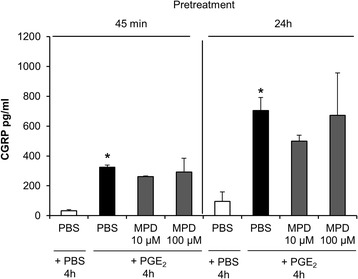
Pretreatment with methylprednisolone (MPD) did not affect PGE_2_-stimulated CGRP release in trigeminal ganglia cell culture. CGRP secretion was determined after pretreatment with PBS or MPD (10 μM or 100 μM) for 45 min or 24 h followed by stimulation with PGE_2_ (10 μM) or vehicle for 4 h. CGRP release was significantly enhanced after PBS + PGE_2_ (* *p* < 0.0001, compared to PBS + PBS). CGRP levels were not altered by pre-stimulation with MPD for 45 min or 24 h (*p* > 0.05, compared to pre-treatment with PBS). CGRP levels are shown in mean pg/ml ± SEM, *n* = 7–8

Subsequently we tested if PGE_2_-induced CGRP release is also altered by pre-stimulation with MPD. There was a statistically significant difference in the CGRP levels between different stimulations as determined by a Kruskal-Wallis H test (χ^2^ (3) = 10.318, *p* = 0.016) Stimulation of cultured trigeminal ganglia cells with PGE_2_ (10 μM) for 4 h led to significantly increased CGRP levels in the supernatant compared to PBS (324 ± 93 vs. 33 ± 7 pg/ml SEM; *n* = 8, *p* < 0.0001). However, the administration of MPD 10 μM (*n* = 8) or 100 μM (*n* = 7) to trigeminal ganglia cells 45 min prior to PGE_2_ stimulation did not alter CGRP release compared to pre-stimulation with vehicle (PBS) (*p* > 0.05). The extension of MPD exposure to 24 h did neither affect PGE_2_-induced CGRP release (*n* = 8) (Fig. 2).

**Fig. 3:**
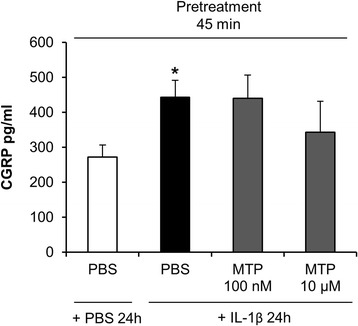
Exposure to metoprolol (MTP) did not affect IL-1β-stimulated CGRP release in trigeminal ganglia cell culture. CGRP secretion was determined 45 min after pretreatment with MTP (100 nM and 10 μM) respectively PBS followed by a 24 h exposure to PBS or IL-1β (10 ng/ml). IL-1β stimulation for 24 h led to a significant CGRP release compared to vehicle stimulation (* *p* = 0.042) which was not altered by pre-treatment with MTP (100 nM or 10 μM) (*p* > 0.05, compared to pre-treatment with PBS). CGRP levels are shown in mean pg/ml ± SEM, *n* = 9

**Fig. 4:**
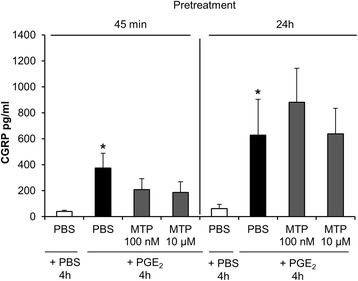
Pretreatment with metoprolol (MTP) did not affect PGE_2_-stimulated CGRP release in trigeminal ganglia cell culture. CGRP secretion was determined 45 min or 24 h after pretreatment with PBS, MTP 100 nM or MTP 10 μM followed by a 4 h exposure to PBS or PGE (10 μM). Stimulation with PGE_2_ resulted in an induction of CGRP release (* *p* < 0.0001 resp. 0.015, compared to treatment with PBS), which was not altered by previous exposure to MTP (100 nM or 10 μM) (*p* > 0.05, compared to pre-treatment with PBS). CGRP levels are shown in mean pg/ml ± SEM, *n* = 9

In a second step we assessed whether the exposure to MTP had any effect on IL-1β- or PGE_2_-induced CGRP release. In contrast to MPD, MTP did not change significantly the amount of stimulus-induced CGRP release in this model. There was a trend towards lower CGRP levels in cultures pretreated with MTP 45 min prior to PGE_2_ exposure. However, the results did not reach statistical significance (*p* = 0.14; *n* = 9), (Figs. 3 and 4). In preliminary experiments (*n* = 4) higher concentrations of MTP (100 μM) did neither alter CGRP release in this model.

## Discussion

In this study MPD blocked IL-1β-induced CGRP secretion in a trigeminal ganglion cell culture model. MPD had no effect on PGE_2_-stimulated CGRP release. Vehicle treated CGRP release in cultured trigeminal ganglia cells was not affected by MPD. The migraine preventive MTP had no effect on IL-1β- or PGE_2_-stimulated CGRP release. In contrast, previous studies in a similar cell culture model showed that the migraine preventives topiramate and botulinum toxin type A blocked CGRP release in cultured trigeminal ganglia cells when stimulated with KCl, capsaicin, protons or nitric oxide [14, 15].

Concentrations and time points for stimulation with IL-1β or PGE_2_ were determined by previous results in this model with maximum CGRP release at these values [8]. Doses for MPD (10 or 100 μM) and MTP (100 nM and 10 μM) used in this experimental study were derived from human data on serum concentrations after applying therapeutically beneficial doses of the drugs. Intake of 80 mg oral methylprednisolone leads to a serum concentration of 7 μM [16]. Intravenous administration of 1000 mg methylprednisolone results in serum concentrations between 16 and 77 μM [17]. Mean serum concentrations of metoprolol after oral application of 100 mg were stated with 136 nM [18]. To assess a maximal effect we chose the higher metoprolol dose equal to the effective dose of methylprednisolone (10 μM).

For pre-stimulation with MTP and MPD we chose 45 min and 24 h before the exposure to PGE_2_ (4 h) to assess the acute effect of stimuli as well as effects that may be mediated through longer acting mechanisms ( e.g. gene expression). Due to the long exposure to IL-1β (24 h) pre-stimulation was restricted to 45 min in these experiments.

Primary trigeminal afferents are a major source of CGRP release into the extracerebral circulation [19]. Activation of trigeminal ganglia afferents and subsequent release of CGRP is thought to play a prominent role in the pathophysiology of migraine [20] and cluster headache [21, 22] pathophysiology. The pro-inflammatory cytokine IL-1β and other cytokines are elevated in migraine [9, 10] and cluster headache patients [11, 23, 24]. The cytokines IL-1β and tumor necrosis factor alpha induce CGRP release in cultured trigeminal ganglia cells [8, 25]. The involvement of immunological mechanisms in primary headaches is possible but the role of cytokines in headache pathophysiology remains incompletely understood.

Cytokines are proteins that are released by glial cells in proximity to peripheral and central neurons. They are involved in pro-inflammatory signaling pathways and represent key elements in the induction and maintenance of pain [26–30]. Increased cytokine expression and pro-inflammatory protein synthesis are both pathophysiological components for the development and maintenance of peripheral and central sensitization. Both mechanisms are important in the pathophysiology of migraine [26, 31, 32]. CGRP itself differentially regulates cytokine secretion from cultured trigeminal ganglion glia cells. In CGRP treated cultures secreted levels of some cytokines (e.g. IL-β) increased while others such as tumor necrosis factor alpha decreased. These results point to a paracrine trigeminal activation due to CGRP release from trigeminal ganglia neurons and glial cytokine secretion that may lead to an inflammatory loop, which could account for sustained sensitization of second-order trigeminal neurons [33]. Chronically sensitized central nociceptive neurons are supposed to contribute to the development of chronic migraine and its resistance to treatment [34].

In contrast to IL-1β-induced CGRP release, PGE_2_-induced CGRP release was not affected by prior exposure to MPD. Previously, we demonstrated that IL-1β induced CGRP release in trigeminal ganglia cells is dependent on COX-2 induction [8]. Methylprednisolone prevents PGE_2_ formation by suppression of COX-2 activity [35]. If inhibition of CGRP release by methylprednisolone is mediated through the prevention of PGE_2_ formation, it is feasible that direct induction of CGRP release by PGE_2_ cannot be blocked by methylprednisolone. In a recent clinical study, we demonstrated the effect of MPD on CGRP release in episodic cluster headache patients in an active bout. A three day pulse therapy with 1000 mg MPD per day led to the normalization of interictally elevated CGRP plasma levels in parallel to the suppression of headache attacks [7]. We extend this observation with our experimental findings in a cell culture of trigeminal ganglia cells. This data support the hypotheses that corticosteroids might exert their preventive action in migraine and cluster headache by the inhibition of trigeminal activation, which is necessary for the initiation of a headache attack. Blockade of trigeminal neurotransmitter secretion could account for prevention of central sensitization and triggering of headache attacks.

Metoprolol, a migraine preventive with slow onset of action had no effect in this experimental model. MTP seems to mediate its prophylactic effect through an alternative mode of action on possible non-inflammatory mechanisms and not on a cellular level in the trigeminal ganglion. The precise mechanism of action of metoprolol in migraine prophylaxis is not known, but modification of cortical excitability by inhibiting central β-receptors most likely contributes to its preventative effects [36–38].

## Conclusions

Pretreatment with MPD blocked IL-1β-, but not PGE_2_-induced CGRP release in cultured primary trigeminal ganglia cells. Based on our findings, we propose that MPD used for short-term cluster headache prophylaxis or prevention of migraine recurrence might act via the suppression of cytokine mediated trigeminal activation.

## Abbreviations

CGRP: Calcitonin gene related peptide
IL-1β: Interleukin-1β
MDP: Methylprednisolone
MTP: Metoprolol
PGE_2_: Prostaglandin E_2_

## References

[1] Uddman R, Edvinsson L, Ekman R, Kingman T, McCulloch J (1985). Innervation of the feline cerebral vasculature by nerve fibers containing calcitonin gene-related peptide: trigeminal origin and co-existence with substance P. Neurosci Lett.

[2] Edvinsson L, Hara H, Uddman R (1989). Retrograde tracing of nerve fibers to the rat middle cerebral artery with true blue: colocalization with different peptides. J Cereb Blood Flow Metab.

[3] Goadsby PJ, Edvinsson L (1994). Human in vivo evidence for trigeminovascular activation in cluster headache. Neuropeptide changes and effects of acute attacks therapies. Brain.

[4] Goadsby PJ, Edvinsson L, Ekman R (1990). Vasoactive peptide release in the extracerebral circulation of humans during migraine headache. Ann Neurol.

[5] Bigal ME, Walter S, Rapoport AM (2013). Calcitonin gene-related peptide (CGRP) and migraine current understanding and state of development. Headache.

[6] Cernuda-Morollon E, Larrosa D, Ramon C, Vega J, Martinez-Camblor P, Pascual J (2013) Interictal increase of CGRP levels in peripheral blood as a biomarker for chronic migraine. Neurology. doi:10.1212/WNL.0b013e3182a6cb72. 10.1212/WNL.0b013e3182a6cb72 23975872

[7] Neeb L, Anders L, Euskirchen P, Hoffmann J, Israel H, Reuter U (2015). Corticosteroids alter CGRP and melatonin release in cluster headache episodes. Cephalalgia.

[8] Neeb L, Hellen P, Boehnke C, Hoffmann J, Schuh-Hofer S, Dirnagl U, Reuter U (2011). IL-1beta stimulates COX-2 dependent PGE synthesis and CGRP release in rat trigeminal ganglia cells. PLoS One.

[9] Perini F, D’Andrea G, Galloni E, Pignatelli F, Billo G, Alba S, Bussone G, Toso V (2005). Plasma cytokine levels in migraineurs and controls. Headache.

[10] Sarchielli P, Alberti A, Baldi A, Coppola F, Rossi C, Pierguidi L, Floridi A, Calabresi P (2006). Proinflammatory cytokines, adhesion molecules, and lymphocyte integrin expression in the internal jugular blood of migraine patients without aura assessed ictally. Headache.

[11] Martelletti P, Granata M, Giacovazzo M (1993). Serum interleukin-1 beta is increased in cluster headache. Cephalalgia.

[12] Huang Y, Cai X, Song X, Tang H, Huang Y, Xie S, Hu Y (2013). Steroids for preventing recurrence of acute severe migraine headaches: a meta-analysis. Eur J Neurol.

[13] Durham PL, Russo AF (1999). Regulation of calcitonin gene-related peptide secretion by a serotonergic antimigraine drug. J Neurosci.

[14] Durham PL, Niemann C, Cady R (2006). Repression of stimulated calcitonin gene-related peptide secretion by topiramate. Headache.

[15] Durham PL, Cady R (2004). Regulation of calcitonin gene-related peptide secretion from trigeminal nerve cells by botulinum toxin type A: implications for migraine therapy. Headache.

[16] Rohatagi S, Barth J, Mollmann H, Hochhaus G, Soldner A, Mollmann C, Derendorf H (1997). Pharmacokinetics of methylprednisolone and prednisolone after single and multiple oral administration. J Clin Pharmacol.

[17] Baylis EM, Williams IA, English J, Marks V, Chakraborty J (1982). High dose intravenous methylprednisolone “pulse” therapy in patients with rheumatoid disease. Plasma methylprednisolone levels and adrenal function. Eur J Clin Pharmacol.

[18] Oosterhuis B, Jonkman JH, Kerkhof FA (1988). Pharmacokinetic and pharmacodynamic comparison of a new controlled-release formulation of metoprolol with a traditional slow-release formulation. Eur J Clin Pharmacol.

[19] Hoffmann J, Wecker S, Neeb L, Dirnagl U, Reuter U (2012). Primary trigeminal afferents are the main source for stimulus-induced CGRP release into jugular vein blood and CSF. Cephalalgia.

[20] Ho TW, Edvinsson L, Goadsby PJ (2010). CGRP and its receptors provide new insights into migraine pathophysiology. Nat Rev Neurol.

[21] Fanciullacci M, Alessandri M, Figini M, Geppetti P, Michelacci S (1995). Increase in plasma calcitonin gene-related peptide from the extracerebral circulation during nitroglycerin-induced cluster headache attack. Pain.

[22] Fanciullacci M, Alessandri M, Sicuteri R, Marabini S (1997). Responsiveness of the trigeminovascular system to nitroglycerine in cluster headache patients. Brain.

[23] Empl M, Forderreuther S, Schwarz M, Muller N, Straube A (2003). Soluble interleukin-2 receptors increase during the active periods in cluster headache. Headache.

[24] Steinberg A, Sjostrand C, Sominanda A, Fogdell-Hahn A, Remahl AI (2011). Interleukin-2 gene expression in different phases of episodic cluster headache--a pilot study. Acta Neurol Scand.

[25] Bowen EJ, Schmidt TW, Firm CS, Russo AF, Durham PL (2006). Tumor necrosis factor-alpha stimulation of calcitonin gene-related peptide expression and secretion from rat trigeminal ganglion neurons. J Neurochem.

[26] Miller RJ, Jung H, Bhangoo SK, White FA (2009). Cytokine and chemokine regulation of sensory neuron function. Handb Exp Pharmacol.

[27] White FA, Jung H, Miller RJ (2007). Chemokines and the pathophysiology of neuropathic pain. Proc Natl Acad Sci U S A.

[28] Uceyler N, Schafers M, Sommer C (2009). Mode of action of cytokines on nociceptive neurons. Exp Brain Res.

[29] Boettger MK, Weber K, Grossmann D, Gajda M, Bauer R, Bar KJ, Schulz S, Voss A, Geis C, Brauer R, Schaible HG (2010). Spinal tumor necrosis factor alpha neutralization reduces peripheral inflammation and hyperalgesia and suppresses autonomic responses in experimental arthritis: a role for spinal tumor necrosis factor alpha during induction and maintenance of peripheral inflammation. Arthritis Rheum.

[30] Schafers M, Sommer C, Geis C, Hagenacker T, Vandenabeele P, Sorkin LS (2008). Selective stimulation of either tumor necrosis factor receptor differentially induces pain behavior in vivo and ectopic activity in sensory neurons in vitro. Neuroscience.

[31] Zhang XC, Kainz V, Burstein R, Levy D (2011). Tumor necrosis factor-alpha induces sensitization of meningeal nociceptors mediated via local COX and p38 MAP kinase actions. Pain.

[32] Yan J, Melemedjian OK, Price TJ, Dussor G (2012). Sensitization of dural afferents underlies migraine-related behavior following meningeal application of interleukin-6 (IL-6). Mol Pain.

[33] Thalakoti S, Patil VV, Damodaram S, Vause CV, Langford LE, Freeman SE, Durham PL (2007). Neuron-glia signaling in trigeminal ganglion: implications for migraine pathology. Headache.

[34] Mathew NT (2011). Pathophysiology of chronic migraine and mode of action of preventive medications. Headache.

[35] Santini G, Patrignani P, Sciulli MG, Seta F, Tacconelli S, Panara MR, Ricciotti E, Capone ML, Patrono C (2001). The human pharmacology of monocyte cyclooxygenase 2 inhibition by cortisol and synthetic glucocorticoids. Clin Pharmacol Ther.

[36] Gerwig M, Niehaus L, Stude P, Katsarava Z, Diener HC (2012). Beta-blocker migraine prophylaxis affects the excitability of the visual cortex as revealed by transcranial magnetic stimulation. J Headache Pain.

[37] Maertens de Noordhout A, Timsit-Berthier M, Timsit M, Schoenen J (1987). Effects of beta blockade on contingent negative variation in migraine. Ann Neurol.

[38] Diener HC, Scholz E, Dichgans J, Gerber WD, Jack A, Bille A, Niederberger U (1989). Central effects of drugs used in migraine prophylaxis evaluated by visual evoked potentials. Ann Neurol.

